# Supplementary material for: Vortex knots in tangled quantum eigenfunctions
Source: Nat Commun. 2016 Jul 29;7:12346. doi: 10.1038/ncomms12346 (PMC4974566; doi:10.1038/ncomms12346)
Supplement: Supplementary Information — Supplementary Figures 1-6, Supplementary Notes 1-4 and Supplementary References [file ncomms12346-s1.pdf]

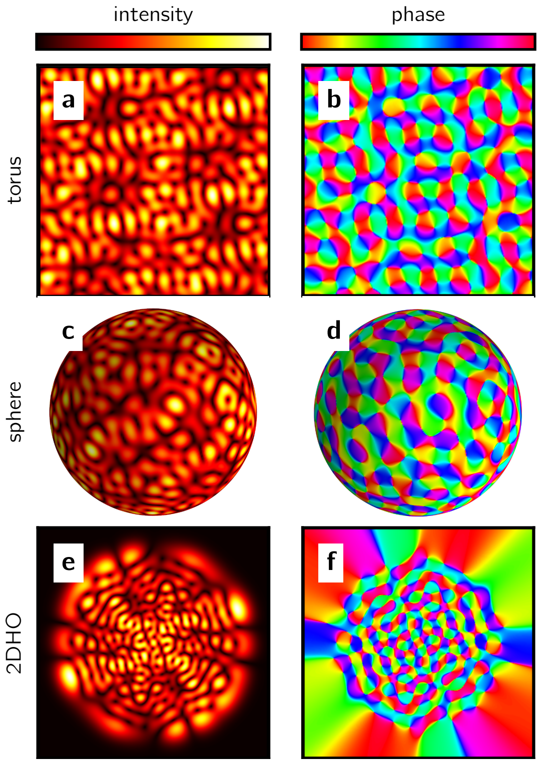

Supplementary Figure 1: The intensity (modulus squared) and phase (argument) of sample random energy eigenfunctions in 2D systems. **a**-**b** square with periodic boundary conditions (flat 2-torus); **c**-**d** the 2-sphere; **e**-**f** the 2D harmonic oscillator (2DHO). These are found using 2D analogues of (1), i.e. complex random superpositions with the same energy (spatial frequency) of 2D plane waves in **a** and **b**, spherical harmonics in **c** and **d**, and 2D harmonic oscillator eigenstates (Hermite-Gauss functions) in **e** and **f**.

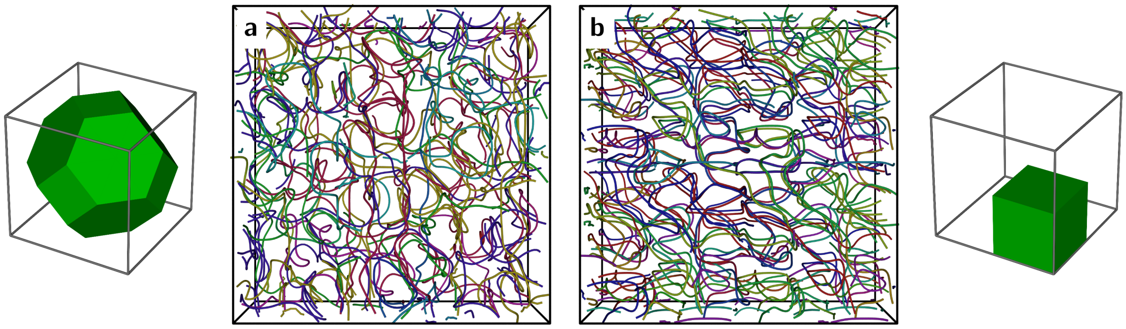

Supplementary Figure 2: Vortices in random energy eigenfunctions in a periodic cubic cell; **a** without and **b** with the additional constraint to octant symmetry. In **a**, $\ell^2 + m^2 + n^2 = 26$ and the periodic unit cell of the vortex tangle is the truncated octahedron. In **b**, $\ell^2 + m^2 + n^2 = 27 = 3 \times 3^2$ and the additional symmetry due to all **k** components being odd means that the periodic unit cell of the vortex tangle is an octant of field's primitive cubic cell.

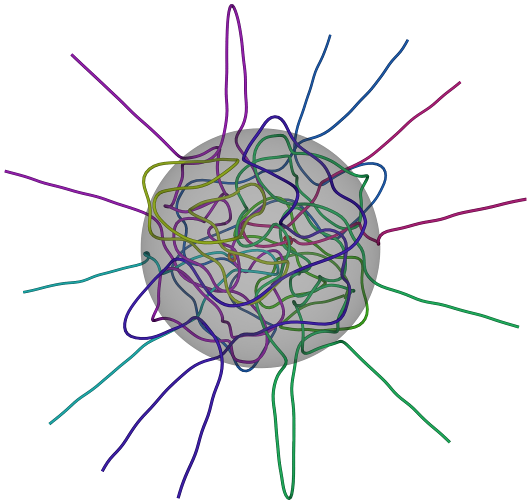

Supplementary Figure 3: Vortices in a random eigenfunction of the 3DHO with N=8. The grey region marks the classical volume where the energy is greater than the potential, and which effectively bounds the vortex tangle; vortices become straight lines as they move further from this shell.

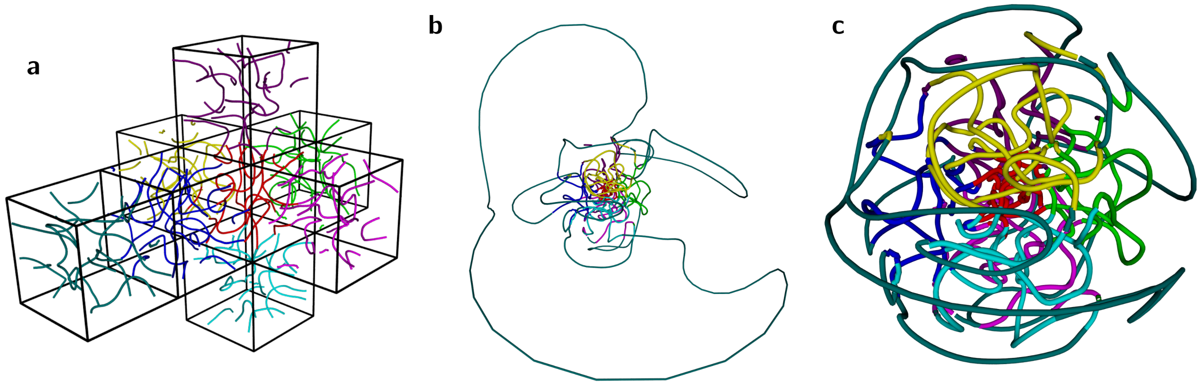

Supplementary Figure 4: Projections of the 3-sphere to 3-dimensional Euclidean space, illustrated with the vortices of a random eigenfunction with $N = 7$. **a** shows the vortices in each octant of a net of the 3-sphere (i.e. a discontinuous map, with the geometry recovered by joining cubic cells along their faces). The vortices within each such octant are given a different colour. In **b** the same vortices are shown via stereographic projection, continuous and angle preserving but significantly distorting distances, with the vortices still coloured according to the octants they passed through in **a**. **c** shows the rescaled stereographic projection used in Figures 1, 3 and 4 of the main text.

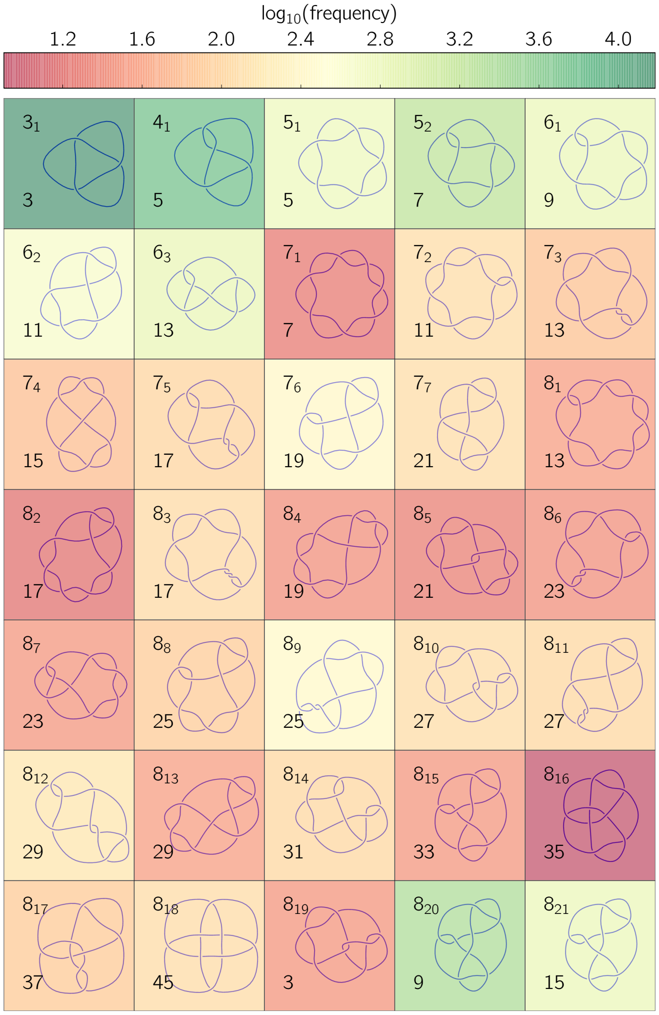

Supplementary Figure 5: The first 35 non-trivial prime knots, ordered by minimal crossing number. These are all the prime knots with 8 or fewer crossings in their minimal projection, with the standard name of the knot (3_1, 4_1, etc) in the top left. The value of the knot determinant $|\Delta(-1)|$ for each knot is also given in the lower left of each cell; of these, none has the same determinant as the unknot (which is unity). The cells are coloured according to the log of its frequency of occurrence in all of our data (unnormalised, and taken over across all systems at various different energies), as detected by the Alexander polynomial at roots of unity. This figure includes a small number of incorrect identifications; the knots 8_{20} and 8_{21} are surprisingly common here, but this is because their Alexander polynomials are equal to those of the more common composite knots 3_1^2 and $3_1\#4_1$ (not depicted). These misidentifications would be corrected by the hyperbolic volume or Vassiliev invariants, which are not applied here. The image of each knot is from the KnotInfo Table of Knot Invariants [19], and the invariant values from KnotInfo and the Knot Atlas [18].

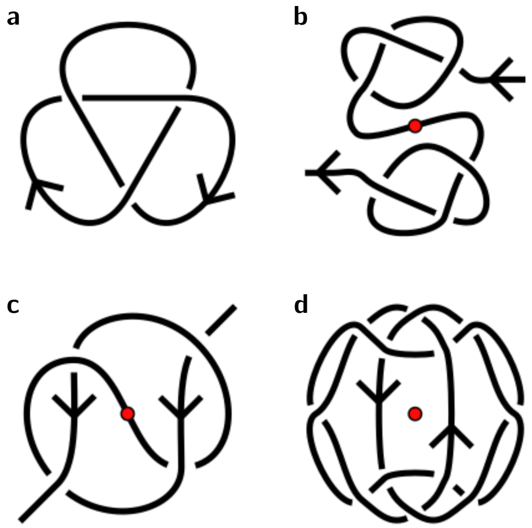

Supplementary Figure 6: Knots exhibiting different symmetries. In **a** a trefoil knot, which is chiral and cannot be transformed to its mirror image without passing the curve through itself. **b**-**d** show strongly amphicheiral conformations of three other knots, equivalent to their mirror images under a rotation by π about the marked red point, and with or without an orientation reversal of the line; in **b** a strongly negatively amphicheiral composite double trefoil knot (3_1^2), in **c** a strongly negatively amphicheiral figure-eight knot (4_1), and in **d** a strongly positively amphicheiral knot 10_{99}.

Supplementary Note 1: Random wave formulation and nodal statistics

The wavefunctions whose nodal structures are considered in the main text are random superpositions of degenerate energy eigenstates in a given system, considered over 3-dimensional position $\mathbf{r} = (x, y, z)$,

$$\psi_j^N(\mathbf{r}) = \sum_j a_j \Psi_j^N(\mathbf{r}) \tag{1}$$

where the sum is over a finite set of indices labelled by j, the a_j are Gaussian random complex variables, and the Ψ_j^N satisfy the time-independent Schrödinger equation $\hat{H}\Psi_j^N = E\Psi_j^N$ for some 3-dimensional Hamiltonian operator $\hat{H}$. Thus $\hat{H}\psi_N = E\psi_N$, and N denotes an integer quantum number.

In the usual random wave model (RWM) which is taken to model wave chaos (for instance, quantum chaotic eigenfunctions in the semiclassical limit) [1, 2], the Hamiltonian is $\hat{H} = -\frac{\hbar^2}{2M}\nabla^2$ for a single particle of mass M at a high energy, so the sum over j is effectively infinite. The ensemble of random functions is statistically isotropic, homogeneous and ergodic, and the (non-normalizable) basis states Ψ_j can be taken to be plane waves with the same spatial frequency $\Psi_j(\mathbf{r}) = \exp(i\mathbf{k}_j \cdot \mathbf{r})$, where $E = \frac{\hbar^2}{2M}|\mathbf{k}_j|^2$.

Unlike the RWM, our numerical realisations are systems involving superpositions over a finite number of degenerate energy eigenfunctions (indexed by principal quantum number N), whose spatial complexity only occupies a finite spatial volume yet whose spatial configuration (including the vortex lines) is statistically similar to the RWM. These systems are the periodic cubic cell, the 3-sphere and the isotropic three-dimensional harmonic oscillator (3DHO). The statistical behaviour of the eigenfunctions of each of these systems approaches that of the isotropic RWM in the limit of high energy. We compare these systems at a range of different energies, from those where knotted vortices first appear to the highest energies practically accessible using the RRCG algorithm introduced in Section . Sample functions of the two-dimensional analogues of these complex random fields are shown in Supplementary Figure 1, where the vortices occur as points (nodes of modulus, phase singularities).

The periodic 3-cell (flat 3-torus) of side length L has the most direct connection to the treatment of the infinite bulk RWM. The Hamiltonian is again $-\frac{\hbar^2}{2M}\nabla^2$ and the eigenfunctions are plane waves with Cartesian components proportional to integers, $\mathbf{k}_j = \frac{2\pi}{L}(\ell, m, n)$. The corresponding energy is $\frac{2\pi^2\hbar^2}{ML^2}(\ell^2 + m^2 + n^2)$, and the degeneracies follow naturally from different triplets of integers having the same sum of squares, with j acting as an index over such triplets.

An extra consideration determines which eigenfunctions of the periodic cell are chosen for our study of vortex tangling. For a typical eigenfunction, the complex field ψ_N is periodic with a cubic fundamental cell. However, it is not difficult to show that for any such ψ, if $\psi(\mathbf{r}) = 0$, then $\psi\left(\mathbf{r} + \frac{L}{2}(1, 1, 1)\right) = 0$ and hence the periodicity of a typical eigenfunction's nodal structure is body-centred cubic. The primitive cell of such a lattice, and therefore of the nodal line tangle, is a truncated octahedron. For simplicity in numerically tracking vortices through the periodic boundaries, we prefer to describe a periodic nodal structure whose primitive cell is a cube. These symmetries of nodal lines in a larger field with cubic symmetry are illustrated in Supplementary Figure 2.

Certain energies give rise to extra symmetries which guarantee this property. When energies are chosen to be $E = \frac{2\pi^2\hbar^2}{ML^2}3N^2$ for integer N, that is, $\ell^2 + m^2 + n^2 = 3N^2$, then ℓ, m, n must be all odd or all even, depending on whether N is odd or even. The nodal structure of a superposition of these plane waves has a primitive cell which is cubic with side length $L/2$, which is an octant of the original cubic cell of the complex wavefunction. It is these smaller cells which are considered in the main text. Energies $E_N \propto 3N^2$ are guaranteed to have at least an eightfold degeneracy with $(\ell, m, n) = (\pm N, \pm N, \pm N)$, and in practice (for sufficiently high N) the degeneracy is much higher. In the examples in the main text, N is chosen to be 9, so triplets of integers whose sum of squares equals 243 are 3,3,15; 5,7,13; 1,11,11 as well as 9,9,9. All together, the total number of plane waves at this energy (counting permutations and all possible signs of components) is 104. The nodal statistics for random eigenfunctions at this energy closely recover local geometrical statistics expected of the isotropic model [3].

In the 3-sphere, coordinates are specified in terms of three angles, χ, θ, ϕ, with $0 \le \chi, \theta \le \pi, 0 \le \phi < 2\pi$. The energy eigenfunctions are those of the (normalised) Laplace-Beltrami operator on the 3-sphere, which are the *hyperspherical harmonics* [4],

$$\mathcal{Y}_{N\ell m}(\chi, \theta, \phi) = \sqrt{\frac{2^{2\ell+1}(N-\ell)!(1+N)}{\pi(1+\ell+N)!}}\, \ell!\, \sin^\ell(\chi)\, C_{N-\ell}^{(\ell+1)}(\cos\chi)\, Y_\ell^m(\theta, \phi)\ , \tag{2}$$

where Y_ℓ^m are the usual spherical harmonics of the 2-sphere, $C_{N-\ell}^{(\ell+1)}$ are *Gegenbauer polynomials*, and for integers N, ℓ, m, $0 \leq N$, $0 \leq \ell \leq N$ and $-\ell \leq m \leq \ell$. The corresponding eigenvalues are labelled by the principal quantum number N, with $E_N = N(N+2)$ (in appropriate units) which are therefore $(N+1)^2$-fold degenerate with the label j corresponding to different values of ℓ and m. In the main text, nodal structures are calculated in these systems for N up to 21. In the stereographic projection of the 3-sphere into spherical polar coordinates, θ and ϕ are the usual spherical angles, and the radial coordinate is $\tan(\chi/2)$.

Random waves in the 3DHO are randomly weighted degenerate eigenfunctions of the Laplacian with an isotropic harmonic potential with angular frequency ω,

$$\hat{H}\psi_N = -\tfrac{\hbar^2}{2m}\nabla^2\psi_N(\mathbf{r}) + \tfrac{m\omega^2}{2}\mathbf{r}^2\psi_N(\mathbf{r}) = E_N\psi_N(\mathbf{r}) \ ,$$

where $E_N = \hbar\omega(N + \frac{3}{2})$ for integer $N \geq 0$. Following the standard theory of the three dimension isotropic harmonic oscillator, the energy eigenfunctions are $\frac{1}{2}(N+1)(N+2)$-fold degenerate [5]. Multiple different bases of energy eigenfunctions can be chosen, but the simplest option for numerical computation utilises Hermite polynomials H_n arising from separation of variables in Cartesian coordinates,

$$\Psi_j^N(\mathbf{r}) = \frac{1}{\sqrt{2^N \ell! m! n!}} \left(\frac{M\omega}{\pi\hbar}\right)^{3/2} H_\ell\left(\sqrt{\tfrac{M\omega}{\hbar}}x\right) H_m\left(\sqrt{\tfrac{M\omega}{\hbar}}y\right) H_n\left(\sqrt{\tfrac{M\omega}{\hbar}}z\right) \exp\left(-\frac{M\omega}{2\hbar}[x^2+y^2+z^2]\right) \ , \quad (3)$$

where j labels triples of nonnegative integers ℓ, m, n such that $N = \ell + m + n$. The resulting vortex tangle is largely confined to within the classical radius $r = \sqrt{2E}$, outside which vortices quickly become geometrically trivial, although they may extend infinitely as shown in Supplementary Figure 3.

In each system, we compute the total vortex length per random eigenfunction. The distribution of these total lengths is found to be numerically strongly peaked at a value given by the integral over the volume of the mean vortex density, which is analogous to calculations of nodal lengths for real random eigenfunctions [6, 7, 8, 9, 10] (the width of the distribution is in each case proportional to the square root of the reciprocal of the degeneracy). Significantly, this total line length is proportional to the systems' total energy E_N. This justifies our comparison in the main text, of energy against knotting probability and complexity, as this provides a physical measure of the total arc length in each tangle. The reference wavelength λ in the main text is proportional to $E_N^{-1/2}$. In all cases, we estimate the numerical error in the calculated total arclength (including that on smoothing the sampled curves) to be no bigger than 5%. Decreasing this would require significantly higher resolution in the sampling, and of course would not affect the topological results.

The vortex density in the ideal isotropic complex RWM is well known to be $2ME/3\pi\hbar^2$ [1, 11]. The other systems approach this limit when $N \gg 1$, but in slightly different ways. The vortex density in the periodic 3-cell depends weakly on direction [3], but at the values of N considered here can be taken to be the isotropic density. The density in the 3-sphere is constant, and can be found using standard methods to be $E_N/3\pi = N(N+2)/3\pi$ (as above, ignoring physical constants and assuming a 3-sphere of unit radius), consistent with the isotropic random wave model result. The vortex density of the 3DHO is the most complicated, as the density is inhomogeneous and isotropic (depending on the value of the radius), and we omit detailed calculations here. Although the detailed results depend subtly (although not strongly) on N, the total arclength we calculate (truncating at twice the classical radius) is indeed found to be proportional to the total energy within comparable error.

In the results of the main text (including Figures 1 and 2), the calculations involved the 3-torus with $N = 9$, with average total arclength approximately 2,000 λ; the 3-sphere with $N = 17$ and average arclength approximately 1,930 λ; and the 3DHO with $N = 21$ and average arclength approximately 1,830 λ. Although these are not exact matches, they are sufficiently close to compare topological statistics which are representative of general trends.

Supplementary Note 2: Numerical techniques

Vortex lines are numerically tracked in 3D complex wavefunctions via a recursively resampled Cartesian grid method (RRCG). The core procedure follows previous numerical experiments [15, 16, 13], sampling the field at points on a 3D Cartesian grid and searching for local 2D grid plaquettes that are penetrated by a vortex. Vortices can be located in this 2D problem either via their intensity (which must be zero) or the circulation of their phase (which must be 2π in a path around the edge of a penetrated plaquette). It is standard to make use of this latter property, as zeros of the intensity are difficult to pinpoint numerically whereas the integrated total change of the phase can be detected even with relatively few sample points situated far from the vortex core; in fact, it is possible to detect most vortex penetrations with just the four sample points at the corners of grid plaquettes with side length around 0.1λ.

A vortex line is additionally *oriented* by the right-handed sense of its quantised phase circulation, and according to this orientation must both enter and leave any grid cell that it passes through (it cannot simply terminate). The above procedure therefore normally detects the passage of a vortex through two different faces of each 3D grid cell. The vortex curve is recovered by joining these points and connecting each line segment with those in neighbouring cells to build up a piecewise-linear approximation to the three-dimensional vortex tangle.

This basic procedure does not perfectly detect vortex curves; vortex penetration of a 2D plaquette may not be detected if the local phase change is too anisotropic on the scale of the lattice spacing. This occurs especially when the lattice spacing is large, or if vortices approach closely, since in this case the 2π integrated phase about multiple vortices cannot be distinguished from the zero phase change which would mean a vortex is not present. Such problems give apparently discontinuous vortex lines, and the numerical procedure *resamples* the complex wavefield in the cells around these apparent discontinuities, generating a new local grid with higher resolution and repeating the search for vortices using this new lattice. If a vortex is discontinuous on the new grid, the resampling procedure is repeated recursively, and is guaranteed to terminate eventually since at very small lengthscales the smoothness of the field limits large phase fluctuations. By matching the different numerical lattices with one another and joining vortices where they pass between them, the recovered vortex curves are continuous within the full numerically sampled region, forming locally-closed loops or terminating on its boundaries. A primary advantage of this method is that it correctly resolves the local topology of vortex lines without requiring a prohibitively high resolution sampling over the entire field. This issue has alternatively been addressed in previous studies using physical arguments [13], an extra random choice [15] or a different grid shape [17], but none of these options is so numerically convenient while guaranteeing robust results. The resampling procedure can also be used to enhance the recovery of local vortex geometry, as described in [3], but this is not important to the topological results described here.

The RRCG algorithm must further be modified in each of the three different systems of wave chaos we consider. With periodic boundary conditions, the finite numerical grid is itself made periodic along each Cartesian axis of the periodic cell, but the RRCG procedure is otherwise unaffected. Vortex loops are recovered by 'unwrapping' vortex segments through the periodic boundaries, equivalent to tiling space with periodic cells and following each loop continuously until its starting point, so the net vortex loop can (and often does) pass through several periodic cell.

In the harmonic oscillator, vortices may extend to infinity and we only consider vortex length within a finite radius of the origin. As distance from the origin increases beyond the classical radius, vortex curves tend to radial lines without further tangling (clearly visible in Supplementary Figure 3, or Figure 1 of the main text), and we take the cutoff at twice the classical radius $\sqrt{2E}$.

Tracking vortices in the 3-sphere is more complicated since it must be projected to flat real space to make it accessible to our 3D Cartesian grid based numerical method. Standard methods such as stereographic projection are numerically inefficient because they greatly distort distances and therefore vortex densities, such that an initial numerical resolution sufficient to detect vortices in the densest regions will be far higher than necessary in other areas where the vortex densities are lower. We instead divide the 3-sphere into a net of eight cubes, with each taken to be a Cartesian grid covering one of the eight octants of the 3-sphere. The RRCG algorithm is run on each octant grid, with overall topology recovered by identification of faces in the overall net. This process is illustrated in Supplementary Figure 4, where 4a shows vortices in each of the cubic octants of the net, discontinuous where they meet the octant faces, while 4b-c show the same vortices in two continuous projections to $\mathbb{R}^3$; these are respectively stereographic projection and the projection used in the main text. Although the spatial round metric of the 3-sphere does vary over each octant, the length variation is in fact relatively small (by no more than a factor of 2) and does not significantly impede vortex tracking efficiency.

Supplementary Note 3: Topological background and techniques

The analysis of topology in wave chaos requires that the topological *knot type* of each vortex curve can be distinguished. It is standard to accomplish this through the calculation of *knot invariants*, which are mathematical objects (integers, polynomials, ...) that can be computed from the geometric conformation but are the same for all representations of the same topological knot (i.e. under ambient isotopy). Mathematical knot theory provides many such invariants, which have been used to develop a taxonomy summarised in *knot tables*, although no invariant is known to distinguish all knots. The values of invariants associated with the *unknot* (i.e. a loop which is not knotted) are usually trivial, whereas the invariants of proper knots usually take other values. Knot tables are usually ordered by the invariant *minimal crossing number*, i.e. according to the smallest number of *crossings* the knot admits on projection into a 2-dimensional plane; the first few prime knots are written 3_1 (the only knot with minimal crossing number 3, i.e. the trefoil knot), 4_1 (the only knot with minimal crossing number 4, i.e. the figure-8 knot), then $5_1, 5_2$, and so on. Supplementary Figure 5 shows the 35 non-trivial prime knots with 8 or fewer minimal crossings. *Composite knots*, those which can be separated into distinct prime knot components each with smaller minimal crossing number such as Figure 1e in the main text, are not included in this table. Composite knots are referred to as combinations of prime knots joined by #, or by exponents for a repeated component; for instance, 3_1^2 for the double trefoil knot or $3_1\#4_1$ for the join of the first two non-trivial knots. Invariants of composite knots can usually be factorised in some sense into those of the component knots, and they are denoted by adjoining the notation of their prime components. Whether a given vortex curve is knotted is found by calculating one or more knot invariants, and then looking these up in the knot table. The values of invariants can also give information about different families and classes of which a given knot is a member [18, 19].

Our primary requirement is to distinguish lines that are knotted from those that are not, and to be able to sort knotted curves by some simple knot invariant which measures their complexity. For this we employ the *Alexander polynomial* [11] $\Delta(t)$, which is straightforward to calculate up to an unknown factor of t^n [12] (e.g. the unknot has Alexander polynomial $\Delta(t) = t^n$, the trefoil knot 3_1 has $\Delta(t) = (1 - t + t^2)t^n$) as the determinant of a matrix with dimension $n - 1$ for projection of the knot with n crossings. Some knotted curves have Alexander polynomial $\Delta(t) = t^n$, like the unknot, but such knots are comparatively rare; the simplest examples are two knots with minimal crossing number 11, already complex enough to be highly uncommon in eigenfunction vortex tangle. Supplementary Figure 5 demonstrates this trend; each knot is coloured by the frequency of its occurrence across all eigenfunction data in all different systems, with most knots by frequency having low minimal crossing numbers, and those with 8 crossings already being up to 1,000 times less common.

Since the projections of curves in our numerical tangle may have several thousand crossings (even after algorithmic simplification), it can be impractical to calculate the full Alexander polynomial symbolically. Thus we evaluate the Alexander polynomial at specific values, conveniently the first three nontrivial roots of unity, -1 (giving the *knot determinant* [12]), $\exp(2\pi i/3)$ and i. The absolute value $|\Delta(t)|$ is invariant under this substitution regardless of the unknown factor of t, and this combination of integer values discriminates the tabulated knots almost as well as the symbolic Alexander polynomial itself; for instance, they are equally discriminatory when distinguishing the 802 prime knots with 11 or fewer crossings. The determinant is a commonly used tool for identifying knots in numerical studies [20, 12], although we have not found Δ at other roots of unity used elsewhere in numerical knot identification.

The knot determinant is also convenient on its own as a measure of knot complexity; it takes its minimum value on the unknot, $\Delta(-1) = 1$, and tends to increase with crossing number (we find it appears on average linearly related to the exponential of the minimal crossing number, consistent with known bounds [21]), and for a composite knot is the product of determinants of its prime components [22]. Many other invariants fulfil these conditions, but the determinant is convenient due to its ease of calculation, the same reason that it is used already in knot detection. Supplementary Figure 5 includes this complexity trend for each of the non-trivial knots with 8 or fewer minimal crossings, taking values from 3 (for the simple trefoil knot 3_1) to 45 (for 8_{18}).

Where it is necessary to distinguish the knot type beyond the discriminatory ability of the Alexander polynomial, such as in Figure 1 of the main text, further invariants are used. Modern knot theory supplies many powerful options, but most of these are impractical to calculate rapidly for large numbers of geometrically complex projections (being calculable only in exponential time), and we instead use more efficient options that are nevertheless sufficient. First, the *Vassiliev invariants* of order two and three are also integer invariants of knots, practically calculable in square or cubic time respectively in the number of crossings of a given diagram, but adding further discriminatory power beyond that of the Alexander polynomial [23]. These invariants have been used previously in numerical knot identification [20]. We also use the *hyperbolic volume*, which takes values in the real numbers and is nonzero only for the so-called

hyperbolic knots, but is highly discriminatory among this class, and for this reason has seen major use in knot tabulation [24]. Most tabulated prime knots are hyperbolic (of the the 1.7 million prime knots with 16 or fewer crossings, only 32 are not [24]), but composite knots always have volume zero and so are also readily separated from prime knots in this way. The hyperbolic volume is calculated using the standard topological manifold routines in SnapPy [25], which return only an approximation but are reliable over the range of complexities we address.

Neither these Vassiliev invariants nor the hyperbolic volume are perfect knot invariants, but combined with the Alexander polynomial they are sufficiently discriminatory to unambiguously identify most simple knots where necessary, such as those in Figures 1 and 4 of the main text. They also further verify that in practice the Alexander polynomial rarely fails to detect knotting among the vortex lines of our eigenfunction systems, as those prime knots with Δ indistinguishable from the unknot are generally easily detected to be hyperbolic.

Supplementary Note 4: Symmetries of knots

Some aspects of knotting in eigenfunctions are dominated by the *symmetry* of the system. Such symmetries have already been removed in our analysis of wave chaos under periodic boundary conditions, but remain in the eigenfunctions of both the 3-sphere and 3DHO.

In the 3-sphere, all eigenfunctions satisfy the condition $\Psi(\psi,\theta,\phi) = (-1)^N\Psi(\pi-\psi,\pi-\theta,\phi+\pi)$ and, since, vortices are nodal lines, a vortex at a given position is always paired with a vortex at its 3-sphere antipode. If these points are on different vortex lines then both lines are identical up to a rotation of the 3-sphere by π through some plane in four dimensions, and so they have the same knot type. If two antipodal points are positions on the same vortex line then the entire vortex curve must be symmetric (carried to itself) under this rotation. Not all knot types can meet this geometric constraint; those that can do so are a subclass of knots that are *strongly amphicheiral*.

Strong amphicheirality is an extension of the more commonly considered chirality of knots; a knot is chiral if it is not equivalent to its mirror image (i.e. with the overstrand and understrand at each crossing switched), and otherwise is called amphicheiral (or equivalently achiral). Both types are common, e.g. the trefoil knot is chiral (shown in Supplementary Figure 6a), but the next non-trivial knot, 4_1, is amphicheiral [26] (evident in the projection of Supplementary Figure 6c, equal to its mirror image under a rotatation by π about the marked point, discussed further below). Strong amphicheirality additionally demands that the knot be equivalent to its mirror not just in its knot type, but under a specific involution of the 3-sphere, i.e. a geometric transformation that is its own inverse [27]. Supplementary Figure 6b-d shows three example diagrams of strongly amphicheiral knots in which the involution is rotation in two dimensions by π about the marked point, which in each case takes the knot diagram to its mirror image; b gives an example of how any composite of a knot with its mirror image admits a strongly amphicheiral conformation [27], c shows strong amphicheirality of the knot 4_1, and d the same for the more complicated 10_{99}.

Strongly amphicheiral knots are additionally split into two classes depending on how the involution affects the orientation of the curve, which for an arbitrary curve may not matter but in vortex lines is fixed by the orientation of the phase. A knot is *strongly negatively amphicheiral* if this orientation is preserved under the involution, or *strongly positively amphicheiral* if its orientation is reversed. Supplementary Figure 6b and 6c show strongly negatively amphicheiral examples (note the reversal of the marked orientation under rotation about the marked point), and this is additionally the reason for these diagrams being drawn as open curves closing at infinity; under the involution of rotation, the origin of rotation is privileged such that the curve must pass through this point and close at infinity (equivalent to the antipode considered on the 3-sphere). No other conformation would be able to meet the strong negative amphicheiral symmetry. In contrast, Supplementary Figure 6d shows a strong positive amphicheiral conformation of the knot 10_{99}. In fact, 10_{99} is the simplest knot with strong positive amphicheiral symmetry, and (with a different conformation) also supports strong negative amphicheirality.

In eigenfunctions of the 3-sphere, the symmetry under rotation reverses the local vortex line orientation according to its phase circulation. This means that such vortices passing through antipodal points can only form strong positive amphicheiral knots, of which the simplest example is the unknot but the first non-trivial prime example is 10_{99}. Although not discussed in the main text, this knot and others with the same symmetry occur with disproportionate frequency in 3-sphere eigenfunctions, with simpler knots occurring only as symmetric antipodal pairs (under the eigenfunction symmetry) or as strong positive amphicheiral composite knots. This symmetry also has an equivalent effect at all N, and so does not lead to significant patterns in knotting probability with energy, as can be seen in Figure 3a of the main text.

Eigenfunctions of the 3DHO have a similar symmetry under inversion through the origin, $\Psi(\mathbf{r}) = (-1)^N\Psi(-\mathbf{r})$, but with the difference now that this supports only strong negative amphicheiral symmetry; the vortex tangent direction is preserved under the inversion. As with the 3-sphere, any vortex line passing through $\mathbf{r}$ is paired with one at $-\mathbf{r}$, and if these points are on different vortices then the entire vortex line appears twice. If the points are on the same vortex line then it must take up a strongly negatively amphicheiral conformation. Under inversion, the only way to do so while meeting the symmetry of the eigenfunction is to pass through the origin and to close at infinity, taking a conformation such as those in Supplementary Figure 6b and 6c. Unlike in the 3-sphere, only one vortex line in a given eigenfunction can do so, and at most one strongly amphicheiral knot can appear. Since the 3DHO naturally supports vortex lines which eventually extend to infinity in straight lines outside the classical radius, it is possible for the privileged origin vortex line to do so and to be knotted.

The probability of a vortex passing through the origin depends directly on N; when N is even, random degenerate eigenfunctions are non-zero at the origin, whereas when N is odd the origin is always a nodal point and sits on a vortex line. This is the reason for the observed odd-even discrepancy in knotting probability with energy in Figure 3 of the main text; when N is even there is no strongly negatively amphicheral vortex line and all knots occur in pairs of antipodal mirrors. When N is odd, such a vortex line always exists and can form a strongly negatively

amphicheiral conformation of a knot. This is relatively common, as the total arclength required to form such a knot is often lower than for the two symmetric copies otherwise required. This knot is frequently prime (unlike with strong positive amphicheirality, there are several strongly negatively amphicheiral knots with fewer than 10 crossings), but can also be a composite of a knot with its mirror image. Compatible knots are thus overrepresented in the statistics of knot type within the system, but now only when N is odd explaining the strong parity dependence of knotting in the 3DHO. The effect is also strong enough to persist even at N high enough that there is a 50% or higher chance for one or more pairs of the other vortex lines in a given eigenfunction to form knots.

Supplementary References

[1] M V Berry & M R Dennis "Phase singularities in isotropic random waves" *Proceedings of the Royal Society A* **456** 2059-79 (2000)

[2] A C White, C F Barenghi, N P Proukakis, A J Youd & D H Wacks "Nonclassical velocity statistics in a turbulent atomic Bose-Einstein Condensate" *Physical Review Letters* **104** 075301 (2010)

[3] A J Taylor & M R Dennis "Geometry and scaling of tangled vortex lines in three-dimensional random wave fields" *Journal of Physics A* **47** 465101 (2014)

[4] H Hochstadt *The Functions of Mathematical Physics* Wiley Interscience (1971)

[5] A I M Rae *Quantum Mechanics* CRC Press 5th edition (2007)

[6] M V Berry "Statistics of nodal lines and points in chaotic quantum billiards: perimeter corrections, fluctuations, curvature" *J Phys A* **35** 3025-38 (2002)

[7] Z Rudnick & I Wigman "On the volume of nodal sets for eigenfunctions of the Laplacian on the torus" *Annales Henri Poincaré* **9** 109-30 (2008)

[8] S Gnutzmann & S Lois "Remarks on nodal volume statistics for regular and chaotic wave functions in various dimensions" *Phil Trans Roy Soc Lond A* **372** 20120521 (2014)

[9] I Wigman "On the distribution of the nodal sets of random spherical harmonics" *J Math Phys* **50** 013521 (2009)

[10] W E Bies & E J Heller "Nodal structure of chaotic eigenfunctions" *J Phys A* **35** 5673-85 (2002)

[11] D Rolfsen *Knots and Links* American Mathematical Society (2004)

[12] E Orlandini & S G Whittington "Statistical topology of closed curves: some applications in polymer physics" *Reviews of Modern Physics* **79** 611-42 (2007)

[13] K O'Holleran, M R Dennis & M J Padgett "Fractality of light's darkness" *Phys Rev Lett* **102** 143902 (2009)

[14] K O'Holleran, M R Dennis, F Flossmann & M J Padgett "Fractality of light's darkness" *Physical Review Letters* **100** 053902 (2008)

[15] T Vachaspati & A Vilenkin "Formation and evolution of cosmic strings" *Phys Rev D* **30** 2036-45 (1984)

[16] B M Caradoc-Davies, R J Ballagh & K Burnett "Coherent dynamics of vortex formation in trapped Bose-Einstein condensates" *Phys Rev Lett* **83** 895-8 (1999)

[17] M Hindmarsh & K Strobl "Statistical properties of strings" *Nucl Phys* **B437** 471-88 (1995)

[18] D Bar-Natan *The Knot Atlas* http://katlas.org (January 2016)

[19] J C Cha & C Livingston *KnotInfo: Table of Knot Invariants*, `http://www.indiana.edu/~knotinfo` (January 2016)

[20] N T Moore, R C Lua & A Y Grosberg "Topologically driven swelling of a polymer loop" *PNAS* **101** 13431-35 (2004)

[21] A Stoimenow "On the coefficients of the link polynomials" *Manuscripta Math* **110** 203-36 (2003)

[22] C E Soteros, D W Sumners & S G Whittington "Entanglement complexity of graphs in Z^3" *Math Proc Camb* **111** 75-91 (1992)

[23] M Polyak & O Viro "Gauss diagram formulas for Vassiliev invariants" *International Mathematics Research Notices* **11** 445-53 (1994)

[24] J Hoste, M Thistlethwaite & J Weeks "The first 1,701,935 knots" *Math Intelligencer* **20** (1998)

[25] M Culler, N M Dunfield & J R Weeks *SnapPy, a computer program for studying the topology of 3-manifolds*, `http://snappy.computop.org` (January 2016)

[26] C C Adams *The Knot Book* American Mathematical Society (1994)

[27] A Kawauchi *A Survey of Knot Theory* Birkhäuser (1996)
